# Epileptic Seizures Induced by a Spontaneous Carotid Cavernous Fistula

**DOI:** 10.1155/2016/9396014

**Published:** 2016-12-19

**Authors:** Güner Koyuncu Çelik, Erkan Yildirim

**Affiliations:** ^1^Faculty of Medicine, Department of Neurology, Baskent University, Ankara, Turkey; ^2^Faculty of Medicine, Department of Radiology, Baskent University, Ankara, Turkey

## Abstract

A 79-year-old woman was admitted to our emergency department with complaints of fainting and loss of consciousness three times during the past month. She was diagnosed with epilepsy and started to be treated with antiepileptic drug. Physical examination showed, in the left eye, chemosis, limited eye movements in all directions, and minimal exophthalmos as unexisting symptoms on admission developed on the sixth day. Orbital magnetic resonance imaging (MRI) and digital subtraction angiography (DSA) imaging revealed a carotid cavernous fistula (CCF). Epileptic attacks and ophthalmic findings previously present but diagnosed during our examinations were determined to ameliorate completely after performing the coil embolization. Based on literature, we present the first case with nontraumatic CCF manifesting with epileptic seizures and intermittent eye symptoms in the present report.

## 1. Introduction

Carotid cavernous fistulas (CCFs) develop as a shunt between the cavernous sinus and carotid arterial system [1]. These fistulas may be classified according to three criteria: direct or indirect (dural) fistulas classified based on angiography and spontaneous or trauma-induced fistulas classified according to their pathogenesis and high-flow and low-flow fistulas based on the hemodynamical nature of the fistula [2]. Type A fistulas diagnosed angiographically are direct connections between the internal carotid artery (ICA) and cavernous sinus, and type B fistulas are between meningeal branches of the ICA and cavernous sinus. In addition, type C fistulas are observed between meningeal branches of the external carotid artery (ECA) and cavernous sinus, and type D fistulas develop between meningeal branches of both ECA and ICA and cavernous sinus [2]. Types B, C, and D fistulas are known as dural or indirect shunts. Spontaneous CCFs are fistulas usually seen in patients with hypertension and mostly developing indirectly and idiopathically in women over 50 years [1].

Spontaneous CCFs may also be related to arteriosclerotic changes of the arterial wall, fibromuscular dysplasia, or Ehler-Danlos syndrome [3]. The clinical presentation of CCFs is related to their size and the flow rate of venous drainage, leading to a variety of symptoms, such as visual loss, proptosis, bruit, chemosis, cranial nerve impairment, intracranial hemorrhages, or infarcts. The treatment modality for CCFs includes endovascular transarterial embolization performed with electrolytically detachable coils, a very effective method with good outcomes. CCFs can also be unilateral or bilateral. Unilateral CCFs can cause bilateral eye symptoms, while bilateral ones can present with unilateral eye symptoms [4]. In other studies, the typical symptoms of CCFs are reported as eye swelling, chemosis, pulsatile exophthalmos, diplopia, and vision loss. Apart from the well-known common symptoms of CCFs, there are also few reports in literature reporting other symptoms of CCFs as isolated cranial nerve palsy, CCF-induced hemispheric laminar necrosis, and brainstem congestion [5–7]. To the best of our knowledge, our report is the first to present a case of epilepsy caused by a spontaneous CCF.

## 2. Case Presentation

**Figure 1:**
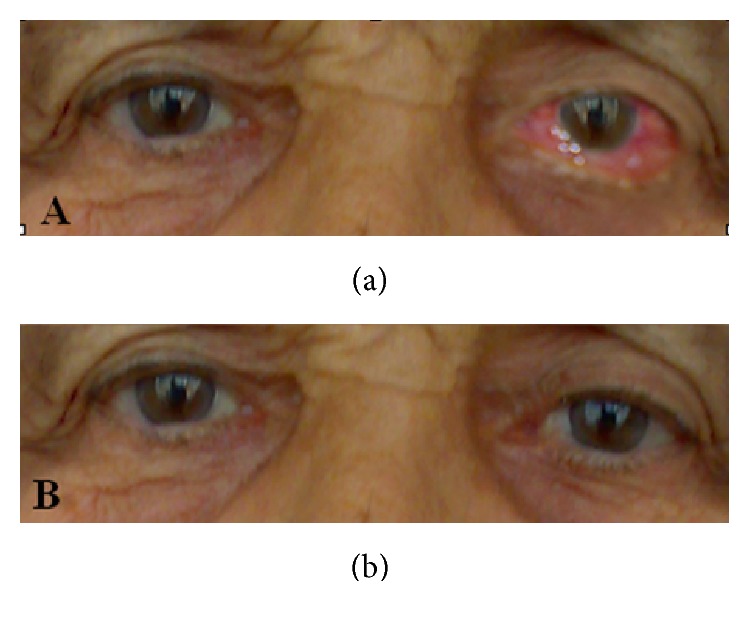
Photographs of the patient. (a) Before the treatment for carotid cavernous sinus fistula showing left chemosis and exophthalmos. (b) After the treatment showing improved chemosis and exophthalmos.

Admitted to the emergency department after the three episodes of fainting and loss of consciousness for one month, our case was a 79-year-old woman maintaining a sedentary life style and living with her family members. The attacks with sudden-onset started with the following signs, blank stares, lip licking, mouth slurping, and tugging clothes, and then continued with convulsions in arms and legs and fainting. The episodes of unconsciousness and fainting lasted for about five to ten minutes. Her history revealed no specific conditions to affect her fainting, such as tiredness, effects led by external factors, fasting, or fainting at a certain moment of the day and neither an additional medical problem nor history in the family for the falling. Neurological and systemic examinations were within normal limits. All laboratory tests and cranial magnetic resonance imaging (MRI) were normal; however, on electroencephalography (EEG) performed while she was awake, slow waves were observed at delta frequency in the left temporoparietal region. The patient was diagnosed with complex partial epilepsy and started to be treated with carbamazepine. Neurological examination showed chemosis, limited eye movements in all directions, and minimal exophthalmos in the left eye on the sixth day, as nonexisting symptoms on admission (Figure 1(a)).

**Figure 2:**
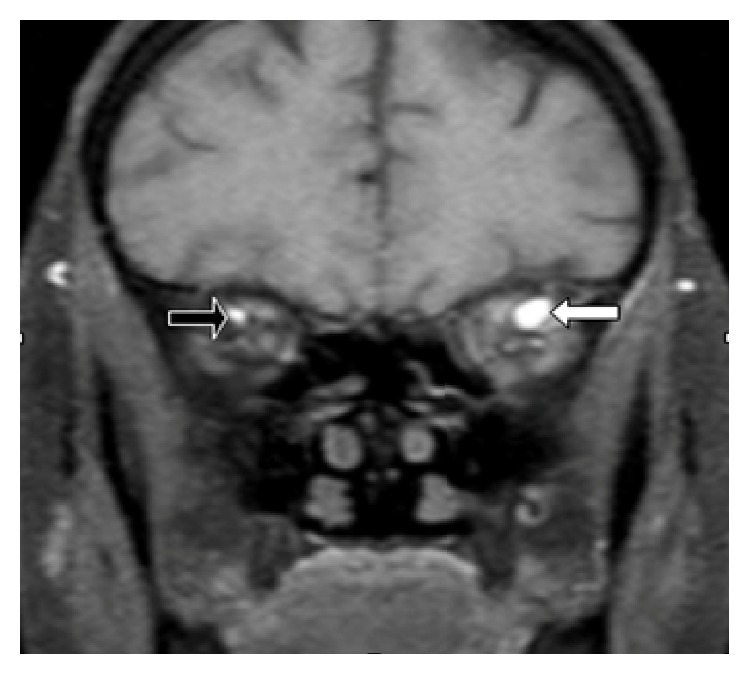
Axial T1-weighed magnetic resonance imaging with contrast demonstrating a greatly dilated left superior ophthalmic vein (white arrow) and normal right superior ophthalmic vein (black arrow).

**Figure 3:**
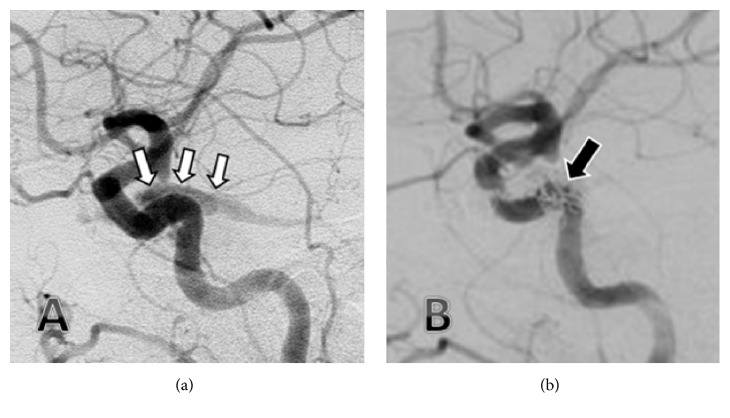
Early arterial phase of the lateral carotid angiography with the filling of the carotid sinus via the fistula ((a) white arrows) and lateral carotid angiogram with the fistula completely closed after transvenous coil embolization ((b) black arrow).

On the eye examination performed by an ophthalmologist on the 6th day, along with exophthalmos, increased intraocular pressure (24 mmHg on the left, 18 mmHg on the right) was detected. Interestingly, on the eye examination performed on the 7th day, the left eye was detected to be completely within normal limits, and all abovementioned symptoms such as chemosis, limited eye movements in all directions, and minimal exophthalmos were seen to disappear. An orbital MRI performed later demonstrated the presence of a dilated left superior ophthalmic vein (Figure 2) and suggested a carotid cavernous fistula (CCF) as the provisional diagnosis. The diagnosis was confirmed through digital subtraction angiography (DSA), and transvenous coil embolization was performed (Figures 3(a) and 3(b)).

All of the symptoms and signs resolved after performing the transvenous coil embolization (Figure 1(b)), and antiepileptic drug treatment was gradually discontinued. On control EEG performed one month after the treatment, the slow waves were seen to disappear. The patient was followed up for three years without any complaints.

## 3. Discussion

In cavernous fistulas, blood passes from the high-pressure arterial system to the low-pressure venous drainage system. This reversal directs a high amount of blood flow to the ophthalmic vein and rarely leads to the cortical venous reflux and the congestion of cortical blood vessels [8]. The venous congestion may cause thrombosis, neural compression [6], or vascular ischemia, alone or in combination [9], and may lead to neurological signs, such as convulsions and stroke [10]. We considered that a fistula developed due to atherosclerosis, and so an epileptic seizure occurred due to the fistula in our case. We observed neither edema nor infarct on cranial MRI, nor thrombosis on DSA. Therefore, we assumed that epileptic seizures were based on neural compression and temporary neural ischemia. The observation of neurological findings without the lesion and the reason why these findings developed intermittently were also considered to arise from neural congestion, compression, and temporary vascular ischemia occurring due to the pressure changes in the fistula. However, the neural congestion, compression, and temporary vascular ischemia due to the pressure of fistula were not shown radiologically due to the insufficiency of medical equipment in our hospital. We consider that the temporary focal slowing we determined on EEG accounts for this condition partly. The most common symptoms of CCF are headache, proptosis, chemosis, murmur, edema, and visual impairment. However, there are few papers in literature reporting rare symptoms as the reasons of CCF such as the hemispheric laminar necrosis and the congestion of the brainstem [2–4], and our report is also one of these rare reports. As well as epileptic attacks, the intermittent ocular symptoms are also encountered rarely in CCF. To the best of our knowledge, there is only one paper in literature reported by Begley et al. and emphasizing that epileptic attacks were seen in CCF. In the case reported by Begley et al., it was recounted that epileptic seizures started 13 months after the trauma, and the cranial computerized tomography revealed brain contusions and edema, as different from our MRI images where no signs were observed [11]. Therefore, it was unclear whether epileptic seizures were due to the fistula or brain trauma. In our case, epileptic seizures were nontraumatic. The intermittent symptoms and epilepsy due to spontaneous nontraumatic fistulas have not yet been reported by any of previous case reports.
